# New Instruments and Appliances

**Published:** 1865-07

**Authors:** 


### NEW INSTRUMENTS AND APPLIANCES.

We some time ago received from S. S. White's, Dental Depot several dental instruments and fixtures, which we had not before used, but having well tested them are free to refer to them. We are always free to do this, in reference to any thing we regard as valuable.

Forbes File Carrier is the best instrument of the kind we have used or seen; all file carriers that have heretofore been introduced were objectionable in some particular. Files are made expressly for it; but any ordinary separating files can be used with it, very thin files can be used in it, with little or no danger of breaking, an accident of very frequent occurrence when used without a carrier.

Bed Plate and Wrench, for Whitiny's vulcanizer, a very convenient arrangement, and one that those who use Whitiny's vulcanizer can not well do without. The plate is fastened to a bench with an opening below it, into which the machine is put to be closed and unclosed.

Trephine for the Antrum. A very useful little instrument for perforating the antrum. This is an operation that, with the ordinary instruments of a dentist's case, is frequently attended with difficulty, but with this instrument is easily accomplished in all cases. There are two sizes.

Dr. Abbott's Pluggers. These are very fine instruments of good shapes, and very perfectly made. They operate finely. A very common fault with both pluggers and excavaturs, is that they are not diffinitely enough formed, and wrought up; these instruments are *certainly very good in this respect.*

